# Effects of β_2_-receptor stimulation by indacaterol in chronic heart failure treated with selective or non-selective β-blockers: a randomized trial

**DOI:** 10.1038/s41598-020-62644-1

**Published:** 2020-04-28

**Authors:** Mauro Contini, Emanuele Spadafora, Simone Barbieri, Paola Gugliandolo, Elisabetta Salvioni, Alessandra Magini, Anna Apostolo, Pietro Palermo, Marina Alimento, Piergiuseppe Agostoni

**Affiliations:** 1 Centro Cardiologico Monzino, IRCCS, Milano, Italy; 2 Department of Clinical Sciences and Community Health, Cardiovascular Section, University of Milano, Milano, Italy

**Keywords:** Cardiology, Drug development

## Abstract

Alveolar β_2_-receptor blockade worsens lung diffusion in heart failure (HF). This effect could be mitigated by stimulating alveolar β_2_-receptors. We investigated the safety and the effects of indacaterol on lung diffusion, lung mechanics, sleep respiratory behavior, cardiac rhythm, welfare, and exercise performance in HF patients treated with a selective (bisoprolol) or a non-selective (carvedilol) β-blocker. Study procedures were performed before and after indacaterol and placebo treatments according to a cross-over, randomized, double-blind protocol in forty-four patients (27 on bisoprolol and 17 on carvedilol). No differences between indacaterol and placebo were observed in the whole population except for a significantly higher VE/VCO_2_ slope and lower maximal P_ET_CO_2_ during exercise with indacaterol, entirely due to the difference in the bisoprolol group (VE/VCO_2_ 31.8 ± 5.9 vs. 28.5 ± 5.6, p < 0.0001 and maximal P_ET_CO_2_ 36.7 ± 5.5 vs. 37.7 ± 5.8 mmHg, p < 0.02 with indacaterol and placebo, respectively). In carvedilol, indacaterol was associated with a higher peak heart rate (119 ± 34 vs. 113 ± 30 bpm, with indacaterol and placebo) and a lower prevalence of hypopnea during sleep (3.8 [0.0;6.3] vs. 5.8 [2.9;10.5] events/hour, with indacaterol and placebo). Inhaled indacaterol is well tolerated in HF patients, it does not influence lung diffusion, and, in bisoprolol, it increases ventilation response to exercise.

## Introduction

β-blockers are a cornerstone therapy in heart failure (HF). Their actions are not limited to the heart but affect several body functions. Indeed, the rearrangement of adrenergic functional signaling in HF is widespread^1–4^. Among the extracardiac effects of β-receptor physiology are those on the lungs, where β-receptors regulate both the bronchial and vascular tone, as well as fluid reabsorption at the alveolar-capillary membrane level. Specifically, β_2_-receptors are located on the alveolar cells, where they regulate the activity of several channels promoting lung fluid clearance^5,6^ Indeed, in HF, a worsening in lung diffusion and exercise capacity has been described after treatment with a non-selective β-blocker, such as carvedilol, in comparison with β_1_-selective β-blockers, such as bisoprolol or nebivolol^7,8^. Recently, some clues of a possible beneficial effect of direct β_2_ alveolar stimulation have been collected as well^9,10^, despite a major concern on the arrhythmic burden of β-stimulation^4^. Moreover, the concomitant presence of systemic β-blockade, especially if non-cardioselective, might interfere with the possible effects of inhaled β-stimulating agents. The aim of our study was therefore to assess the efficacy and safety of a 2-month treatment with an inhaled β_2_ agonist in HF patients on treatment with a β_1_-selective (bisoprolol) or with a non-selective (carvedilol) β-blocker. The main endpoints were change in quality of life, arrhythmic burden, lung mechanics, lung diffusion, aerobic exercise capacity, and sleep respiratory disorders. Among the different β_2_-receptor stimulating agents, we chose indacaterol because it is a highly β_2_-selective, well tolerated agent with a strong safety profile.

## Methods

### Study population

This is a single-center, randomized, double-blind, prospective, cross-over study on the effects of indacaterol in stable HF patients treated with a β-blocker, performed in two parallel arms according to β-blocker therapy (carvedilol or bisoprolol).

Study inclusion criteria were age >18 years, chronic HF with reduced systolic function (left ventricular ejection fraction − LVEF − <40%), stable clinical conditions, stable and optimized pharmacological therapy for at least two months, including β-blockade with either carvedilol or bisoprolol, mild chronic obstructive lung disease (COPD) demonstrated by a forced expiratory volume in 1 s (FEV_1_)/vital capacity (VC) < 100% of the predicted value, never having been treated with bronchodilator compounds.

Exclusion criteria were history and/or clinical documentation of pulmonary embolism or primary valvular heart disease, pericardial disease, severe obstructive or restrictive lung disease, asthma or use of bronchodilators, primary pulmonary hypertension, severe renal failure (eGFR < 30 ml/min/1.73 m^2^), significant peripheral vascular disease, second or higher degree atrioventricular block at EKG, exercise-induced angina and/or ischemic ST changes and/or repetitive ventricular arrhythmias, severe ventricular arrhythmias at 24-hour Holter monitoring, uncontrolled systemic hypertension, epilepsy or convulsive disorders, uncontrolled diabetes (HBA1c > 8% of total hemoglobin), evidence or history of long QT syndrome (specifically, patients with a QTc calculated by Fridericia formula >450 msec for males or >470 msec for females at run-in were excluded), concomitant use of steroids, sympathomimetic drugs or strong or moderate inhibitors of CYP3A4 or P Glycoprotein, such as amiodarone. We also excluded patients not able to adequately perform pulmonary function tests and/or diffusing capacity test, not able/willing to complete a maximal cycle ergometer cardiopulmonary exercise test (CPET), and patients with cardiac resynchronization therapy, hemodynamic, electrophysiological, or surgical procedures planned in the following four months.

The protocol was approved by the local ethics committee. All subjects gave their written informed consent (“Indacaterol in Heart Failure Patients: Any Role on Lung Fluid Regulation?”, Trial registration November 6, 2015, Clinical Gov Trials number: NCT02598505 EudraCT: 2014-001360-35).

### Study procedures

At every step of the study protocol (see study design section), a 12-lead electrocardiogram (EKG) was recorded for each patient, in supine position after 5 minutes of quiet rest, by which resting heart rate (HR) and QTc (Fridericia) were calculated. At the same time, rest blood pressure was measured, a blood venous sample was collected for detection of hemoglobin, urea, creatinine, potassium, glucose, and BNP plasma concentrations, and a questionnaire for life quality evaluation was administered (Minnesota Living With HF Questionnaire – MLWHFQ). Afterwards, the following diagnostic tests were performed:

#### Pulmonary function test with lung diffusion measurements

Standard pulmonary tests were performed according to the American Thoracic Society criteria^11^. The normal predicted values for FEV_1_ and VC were those reported by Quanjer *et al*.^12^. Lung diffusion for carbon dioxide (DL_CO_) and for nitric oxide (DL_NO_) were simultaneously measured in the standard sitting position through the single-breath technique, with a breath-hold time of 4 seconds (MS-PFT analyzer, Jaeger Masterscreen, Hoechberg, Germany). Membrane diffusion (D_M_) subcomponent was calculated dividing DL_NO_ by 1.97, while capillary volume (Vc) was estimated from DL_CO_ and D_M_ through Roughton and Foster’s formula^13^. Alveolar volume (V_A_) was measured by helium decay slope during single-breath constant expiratory flow measurement.

#### CPET

Maximal CPETs were performed on a cycle ergometer (Ergo 800S, Sensor Medics, Yorba Linda, CA) applying a ramp protocol personalized for each patient, designed to reach maximum exercise in about 10 ± 2 minutes^14^. All patients performed at least one familiarization procedure before the protocol run-in. Patients breathed into a mass flow sensor through a mouthpiece connected to a saliva trap and wearing a nasal clip or through a facial mask, according to their preference and to facial and dental conformation. Ventilation (VE), oxygen consumption (VO_2_), and carbon dioxide production (VCO_2_) were measured breath by breath (V-max 2900 metabolic cart, Sensor Medics, Yorba Linda, CA). HR and 12-lead EKG were monitored continuously, hemoglobin saturation was recorded by an ear oximeter, and blood pressure was monitored with a cuff sphygmomanometer every 2 minutes. Anaerobic threshold (AT), VE/VCO_2_ slope, and VO_2_ vs. work relationship were measured following a standard methodology^15^.

#### Twenty-four-hour Holter EKG recording

Standard ambulatory 24-hour Holter EKG recording was performed through Pathfinder system (Pathinder Digital, Spacelabs Healthcare, Snoqualmie, Washington, USA).

#### Nocturnal cardiorespiratory monitoring (NCM)

Sleep respiratory behavior was recorded by a portable system (Embletta x100, SapioLife S.r.L., Monza, Italy) with simultaneous and continuous recording of respiratory flow and snoring by a nasal cannula, thoracic and abdominal respiratory effort by strain gauges, and oxygen saturation (SaO_2_) by a finger digital oximeter. Apneas were defined as a reduction in the amplitude of respiratory flow signal below 10% of the baseline value for at least 10 seconds, while hypopneas were defined as a reduction of respiratory flow between 10 and 50% of the baseline for at least 10 seconds associated with an oxygen desaturation of at least 3%. Apneas were considered of central origin when the interruption in respiratory flow was associated with absence of thoracic and abdominal respiratory effort, obstructive if a respiratory thoracic or abdominal activity was present during the stop in respiratory flow, and mixed when an initially central apnea turned into obstructive in its terminal phase^16^. Hypopneas were not further classified. Apnea-hypopnea index (AHI) was defined as the number of apneas and hypopneas per hour of time in bed (defined as the time spent between light switch off and switch on, in recumbent position and without major body movements). Central apnea, mixed apnea, obstructive apnea, and hypopnea indexes were calculated with the same method as AHI.

### Study design

**Figure 1:**
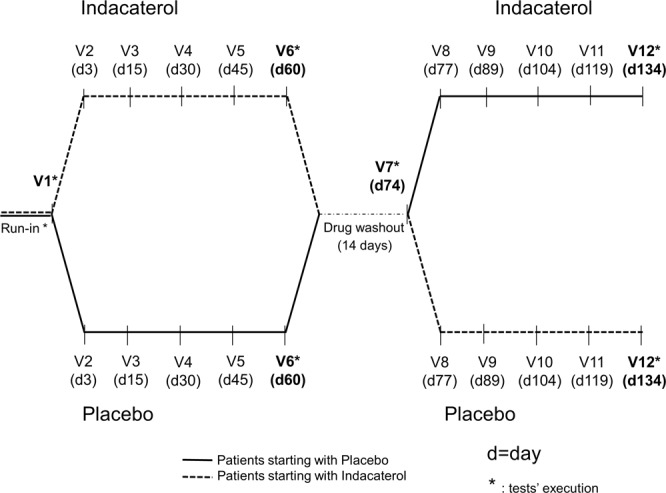
Study design.

All consecutive patients fulfilling inclusion and exclusion criteria and who gave their written informed consent to the study were enrolled, and they performed a maximal CPET to identify a personalized ramp protocol. The day before starting the experimental drug administration, a 24-hour Holter EKG recording and an NCM were performed. On day one of the study (V1), procedures other than Holter EKG and NCM were performed as described above. Holter EKG data were immediately evaluated to exclude subjects with severe ventricular arrhythmias. After completion of all tests, patients were blindly randomized 1:1 to indacaterol or placebo treatment for a two-month period, planning the first follow-up examination after 3 days and the following every 15 days to check for drug compliance and tolerability and for adverse events. After two months of treatment (V6), patients performed all the study procedures. After a 14-day wash-out of the experimental drug, patients returned to the research laboratory (V7) to repeat the same study procedures and to restart treatment with the experimental drug according to a cross-over design: patients taking placebo in the V1-V6 period shifted to indacaterol treatment, and vice versa. After another two-month treatment period with examinations planned at the same intervals as in the V1-V6 period, all patients performed the study procedures again (V12), and the study was considered completed (Fig. 1). At V2, V3, V4, V5, V8, V9, V10, and V11, patients underwent clinical evaluation, and compliance to study protocol was assessed. In order to maintain the double blindness, an external firm produced identical capsules and identical packaging for both placebo and indacaterol. Blister packs were identified by the expression “period 1” (administered between V1 and V6) and “period 2” (administered between V7 and V12), containing placebo or indacaterol, following a predetermined randomization list obtained with a pre-specified software (PMX CTM 3.2/(c) Propack Data GmbH, NerPharMa) with 25 blocks (block size = 4). Both patients and physicians were blinded to the content of the blister packs.

The primary endpoints of the study were the changes in lung diffusion, expressed by DL_CO_, after indacaterol treatment compared with placebo treatment in the whole population and in the bisoprolol and carvedilol groups, and the safety of indacaterol in the whole population as evaluated by clinical, EKG, 24-hour Holter EKG, and blood chemistry parameters.

### Statistical analysis

A population sample of 60 patients (30 for each β-blocker group) was planned in order to detect a difference in the primary endpoint (DL_CO_ difference between indacaterol and placebo) of at least 2 ± 1.5 ml/min/mmHg with a power >95%. Continuous variables are presented as mean ± SD, while non-normally distributed variables are presented as median and interquartile ranges (IQR). The analyses to evaluate differences between the indacaterol and the placebo group were performed using paired t-test for AB/BA cross-over design^17^. Subsequently, a subgroup analysis was implemented to assess the potential difference between drug and placebo groups within and between the two β-blockers, yet again with paired t-test for AB/BA cross-over design. All calculations were computed with the aid of the SAS software package (Version 9.4 SAS Institute Inc., Cary, NC).

## Compliance with Ethical Standards

### Research involving human participants and/or animals

This research involves humans. This article does not contain any studies with animals performed by any of the authors.

### Ethical approval

All procedures performed in studies involving human participants were in accordance with the ethical standards of the institutional research committee (ethics committee “Centro Cardiologico Monzino” approved the protocol with number: CCM89) and with the 1964 Helsinki declaration and its later amendments or comparable ethical standards.

### The protocol is registered at clinical Gov trials

NCT02598505 EudraCT: 2014-001360-35.

### Informed consent

Informed consent was obtained from all individual participants included in the study.

## Results

All eligible patients referring to the HF Unit of Centro Cardiologico Monzino between September 2015 and September 2016 were consecutively evaluated for participating in the study, and 48 patients gave their written informed consent to the study protocol and begun the study treatment and procedures. One patient died during follow-up because of cardiac arrest as a consequence of HF worsening during the placebo treatment period. Four patients experienced serious adverse events: one during placebo (hospitalization because of systemic inflammatory disease), 2 during indacaterol treatment (paroxysmal atrial fibrillation and acute bowel obstruction, respectively) and one in the wash-out period (transient cerebral ischemia few days after the end of the indacaterol treatment period). One patient reported a non-severe adverse event during placebo (dyspnea). Only one adverse event (the atrial fibrillation episode) was considered to be possibly related to the experimental drug. Globally, 4 out of 6 patients (2 on placebo and 2 on indacaterol) who reported adverse events discontinued the experimental drug and did not complete the study procedures (one because of death and three having withdrawn their informed consent after discussion with the medical team about the risk of study prosecution), while 2 patients continued the research protocol. Patient enrollment was prematurely interrupted after the inclusion of 48/60 patients because of the unavailability of placebo doses 12 months after the beginning of the study. In the end, 44 patients completed all study procedures through the whole study period and were included in the analysis, 27 of them were treated with bisoprolol and 17 with carvedilol. The baseline characteristics of the entire study population are reported in Table 1. No significant differences were observed between patients receiving carvedilol and bisoprolol except for a significantly higher rest HR and an almost significantly lower LVEF in the carvedilol group (respectively HR 70 ± 10 vs. 63 ± 10 bpm, p < 0.03, and LVEF 31.2 ± 6.8 vs. 34.6 ± 4.6%, p = 0.051) (Table in Supplementary Information).

**Table 1:** Patients characteristics (n = 44).

Variable		Mean ± SD n/%
Age (years)		66.04 ± 9.61
Gender (Males N/%)		31/70%
Smoking habit	No smokers (N/%)	17/39%
	Former smokers (N/%)	27/61%
Rhythm	Sinus rhythm (N/%)	37/84%
	Atrial fibrillation (N/%)	7/16%
Mitral regurgitation	Mitral regurgitation absent (N/%)	15/34%
	Mitral regurgitation mild (N/%)	19/43%
	Mitral regurgitation moderate (N/%)	9/21%
	Mitral regurgitation severe (N/%)	1/2%
Drugs	ACE inhibitors (N/%) No	17/39%
	ACE inhibitors (N/%) Yes	27/61%
	ATII blockers (N/%): No	32/73%
	ATII blockers (N/%): Yes	12/27%
	Diuretics (N/%) No	8/18%
	Diuretics (N/%) Yes	36/82%
	Antialdosteronics (N/%) No	10/23%
	Antialdosteronics (N/%) Yes	34/77%
	Digitalis (N/%) No	41/93%
	Digitalis (N/%) Yes	3/7%
Systolic Arterial Pressure (mmHg)		123 ± 17
Diastolic Arterial Pressure (mmHg)		76 ± 8
	Aetilogy: ischemic	23/52%
	Aetilogy: idiopathic	19/43%
	Aetilogy: other	2/5%
Body mass index		28.1 ± 4.6
Left Ventricular Ejection Fraction (%)		33.3 ± 5.7
Rest heart rate (bpm)		66 ± 10
Haemoglobin (g/dl)		13.8 ± 1.3
BNP (pg/ml)		291.3 ± 249.5
MDRD (ml/min/1.73 mq)		71.8 ± 21.0
NYHA class	NYHA class I	7/16%
	NYHA class II	31/70%
	NYHA class III	6/14%
MLWHFQ Score		28.4 ± 20.0
FEV1 (L/min)/(% predicted value)		2.34 ± 0.71/84.5 ± 14.0
VC (L)/(% predicted value)		3.28 ± 0.95/91.9 ± 14.1
DLco (ml/min/mmHg)		19.91 ± 6.13
Peak VO_2_ (ml/Kg/min)		14.6 ± 3.6

NYHA = NewYork Heart Association; BNP = Brain Natriuretic Peptide; MDRD = estimation of glomerular filtration rate by modification of diet in renal disease formula; FEV1 = Forced Expiratory Volume in 1 second; VC = Vital Capacity; DLco = Lung diffusion for carbon monoxide; VO_2_ = Oxygen consumption; MLWHF = Minnesota Living With Heart Failure.

Indacaterol-related effects were assessed comparing active drug vs. placebo (V6 and V12, Fig. 1). In Table 2, data related to treatment safety are reported, derived from clinical evaluation, 12-lead EKG, blood samples, and 24-hour Holter EKG in the whole population. No differences were observed in any of the investigated parameters after indacaterol treatment in comparison to placebo, with the only exception of maximum HR recorded during 24-hour Holter EKG, which appeared to be significantly higher with indacaterol.

**Table 2:** Comparison of safety parameters after treatment with placebo or indacaterol in the whole study population.

		Placebo	Indacaterol	p
	**NYHA Class**	1.9 ± 0.6	1.8 ± 0.5	0.2623
	**SBP** (mmHg)	122 ± 16	128 ± 21	0.1901
	**DBP** (mmHg)	76 ± 7	76 ± 10	0.7752
24-hours Holter Recording	**Mean HR** (bpm)	65 ± 9	66 ± 10	0.7098
	**Maximum HR** (bpm)	95 ± 14	102 ± 24	**0.0321**
	**Minimum HR** (bpm)	54 ± 6	54 ± 9	0.3843
	**VE** (n/hour)	32.5 (11;128)	17.5 (5;85)	0.9721
	**SVE** (n/hour)	1.4 (0;4)	1.4 (0;5)	0.5000
	**Couples** (n)	3 (0;37)	4 (0;43)	0.9442
	**SV Run** (n)	0 (0;0)	0 (0;0)	0.8811
	**V Run** (n)	0 (0;1)	0 (0;1)	0.3224
	**Triplettes** (n)	0 (0;0)	0 (0;1)	0.2927
Blood Sample	**Glucose** (mg/dl)	127 ± 43	124 ± 36	0.4560
	**Creatinine** (mg/dl)	1.1 ± 0.3	1.12 ± 0.3	0.3725
	**BNP** (pg/ml)	170 (50;423)	174 (60;358)	0.1578
	**Hb** (g/dl)	13.9 ± 1.4	13.9 ± 1.5	0.1028
	**MLWHF Score**	15 (7;28)	12 (4;27)	0.3079

NYHA = NewYork Heart Association; SBP = Systolic Blood Pressure; DBP = Diastolic Blood Pressure; HR = Heart Rate; VE = Ventricular Ectopies; SVE = SupraVentricular Ectopies; SV = SupraVentricular; V = Ventricular; BNP = Brain Natriuretic Peptide; Hb = Haemoglobin; MLWHF = Minnesota Living With Heart Failure.

**Figure 2:**
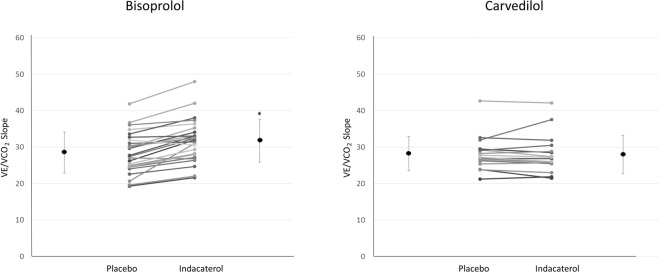
Changes in VE/VCO_2_ slope from placebo to indacaterol treatment in the bisoprolol and carvedilol groups. *p < 0.0001.

In Tables 3–5, the effects of indacaterol treatment on lung mechanics, lung diffusion, exercise, and nocturnal respiratory behavior are reported in the entire population and separately in the carvedilol and bisoprolol groups. No significant difference was observed in the whole study population between indacaterol and placebo treatments for any of the explored parameters, except for a significant increase in VE/VCO_2_ slope (Fig. 2) and a reduction in maximal P_ET_CO_2_ after indacaterol, and a significant, but clinically negligible, longer snoring time with indacaterol treatment. Similarly, no significant effect of indacaterol vs. placebo was observed in the carvedilol group, with the only exception of a significant reduction in the hypopnea index. Conversely, in the bisoprolol group, a trend was observed towards higher vital capacity and FEV_1_ and a significantly greater ventilatory response to exercise (higher VE/VCO_2_ slope) and lower maximal P_ET_CO_2_ after indacaterol as compared to placebo. A significant inverse correlation was observed as well between the changes in maximal P_ET_CO_2_ and VE/VCO_2_ slope after indacaterol compared to placebo in the whole population (R −0.48, p < 0.002).

**Table 3:** Comparison of lung mechanics and lung diffusion parameters after treatment with placebo or indacaterol in the whole study population and in the cardvedilol and bisoprolol groups.

	All patients			Bisoprolol			Carvedilol		
	Placebo	Indacaterol	p	Placebo	Indacaterol	p	Placebo	Indacaterol	p
**VC** (L)	3.24 ± 0.96	3.30 ± 0.96	0.2001	3.15 ± 1	3.26 ± 1	0.1645	3.38 ± 0.9	3.36 ± 0.9	0.9721
**FEV1** (L)	2.28 ± 0.73	2.36 ± 0.69	**0.0778**	2.27 ± 0.8	2.35 ± 0.7	**0.0628**	2.3 ± 0.6	2.37 ± 0.6	0.5953
**AV** (L)	4.54 ± 1.0	4.56 ± 0.9	0.887	4.44 ± 1	4.55 ± 0.9	0.231	4.71 ± 0.9	4.57 ± 0.9	0.0616
**DLco** (ml/min/mmHg)	18.99 ± 5.5	19.5 ± 6.2	0.7878	18.33 ± 5.7	18.6 ± 6.5	0.5864	20.03 ± 5.2	20.82 ± 5.7	0.4217
**DLco/AV** (ml/min/mmHg)	4.18 ± 0.9	4.16 ± 1.2	0.3187	4.13 ± 1	3.89 ± 1.2	0.2815	4.26 ± 0.8	4.56 ± 1	0.9126
**DM** (ml/min/mmHg)	29.36 ± 10.3	29.81 ± 10.6	0.4336	27.97 ± 10.3	28.19 ± 11.1	0.6905	31.58 ± 10	32.29 ± 9.5	0.5315
**DM/VA** (ml/min/mmHg)	6.36 ± 1.2	6.48 ± 1.7	0.7242	6.2 ± 1.2	6.15 ± 1.9	0.7183	6.61 ± 1.2	6.99 ± 1.2	0.8671
**Vc** (mL)	65.38 ± 32	71.19 ± 35.5	0.4775	61.68 ± 30.4	67.6 ± 38.2	0.5971	71.04 ± 34.5	76.26 ± 31.8	0.6188
**DLNO**	71.06 ± 24.9	73.54 ± 23	0.7091	67.69 ± 25	70.54 ± 22.9	0.8238	76.42 ± 24.3	78.13 ± 23.1	0.9749

VC = Vital Capacity; FEV1 = Forced Expiratory Volume in 1 second; AV = Alevolar Volume; Dlco = Lung diffusion for carbon monoxide; DM = membrane conductance; Vc = Capillary Volume; DLNO = Lung Diffusion for Nitric Oxide.

**Table 4:** Comparison of cardiopulmonary exercise test parameters after treatment with placebo or indacaterol in the whole study population and in the cardvedilol and bisoprolol groups.

	All patients			Bisoprolol			Carvedilol		
	Placebo	Indacaterol	p	Placebo	Indacaterol	p	Placebo	Indacaterol	p
**Peak VO**_**2**_ (ml/min)	1188 ± 451	1219 ± 425	0.2196	1148 ± 425	1153 ± 385	0.4467	1253 ± 494	1325 ± 474	0.3816
**Peak VCO**_**2**_ (ml/min)	1396 ± 583	1429 ± 515	0.1699	1375 ± 565	1370 ± 478	0.7245	1428 ± 627	1522 ± 570	0.2128
**Peak VE** (L/min)	50.9 ± 18.2	51.3 ± 13.8	0.1391	51.3 ± 19.3	50.5 ± 13.3	0.3826	50.2 ± 16.9	52.5 ± 14.8	0.2613
**Peak Vt** (L/min)	1.57 ± 0.6	1.58 ± 0.5	0.2116	1.55 ± 0.6	1.52 ± 0.5	0.9225	1.6 ± 0.5	1.68 ± 0.5	0.1566
**Peak RR** (acts/min)	33 ± 6	33 ± 6	0.3775	34 ± 7	34 ± 6	0.1798	32 ± 6	32 ± 5	0.9332
**Peak HR** (bpm)	110 ± 24	113 ± 26	0.1198	109 ± 20	110 ± 19	0.9232	113 ± 30	119 ± 34	0.0357
**Peak RER**	1.16 ± 0.1	1.17 ± 0.1	0.3324	1.18 ± 0.1	1.19 ± 0.1	0.9192	1.13 ± 0.1	1.14 ± 0.1	0.2108
**Peak O**_**2**_ **pulse** (ml/beat)	10.9 ± 3.5	11 ± 3.6	0.5040	10.7 ± 3.8	10.8 ± 3.9	0.4694	11.1 ± 3.2	11.3 ± 3.2	0.8766
**Peak Work** (Watt)	82 ± 41	83 ± 37	0.3868	77 ± 38	77 ± 33	0.9132	90 ± 45	92 ± 42	0.3353
**AT VO**_**2**_ (ml/min)	786 ± 281	810 ± 293	0.5557	745 ± 243	760 ± 262	0.8977	848 ± 328	887 ± 329	0.4005
**VO**_**2**_**/Work Slope** (ml/min/watt)	9.9 ± 1.3	9.9 ± 1.5	0.8436	9.7 ± 1.2	9.7 ± 1.7	0.9365	10.2 ± 1.3	10.2 ± 1.3	0.9027
**VE/VCO**_**2**_ **Slope**	28.4 ± 5.2	30.1 ± 5.6	**0.0008**	28.5 ± 5.6	31.8 ± 5.9	**<0.0001**	28.2 ± 4.7	27.7 ± 4.2	0.8752
**P**_**ET**_**CO**_**2**_ **Maximal** (mmHg)	37.9 ± 5.1	37.3 ± 5.0	**0.0026**	37.7 ± 5.8	36.7 ± 5.5	**0.0148**	38.1 ± 4.1	38.1 ± 4.3	0.4916
**P**_**ET**_**CO**_**2**_ **Peak** (mmHg)	33.6 ± 5.5	33.3 ± 5.3	0.1495	33.6 ± 6.3	32.9 ± 5.5	0.1142	33.6 ± 4.3	34.0 ± 5.1	0.5159

VO_2_ = Oxygen Consumption; VCO_2_ = Carbon Monoxide production; VE = Ventilation; Vt = tidal Volume; RR: Respiratory Rate; HR = Heart rate; RER = Respiratory Exchange Ratio; AT = Anaerobic Thresold, PETCO_2_ = maximal end-tidal CO_2_ partial pressure.

**Table 5:** Nocturnal cardiorespiratory monitoring parameters after treatment with placebo or indacaterol in the whole study population and in the cardvedilol and bisoprolol groups.

	All patients			Bisoprolol			Carvedilol		
	Placebo	Indacaterol	p	Placebo	Indacaterol	p	Placebo	Indacaterol	p
**TIB** (min)	428 ± 77	439 ± 79	0.2745	422 ± 62	444 ± 56	0.3571	426 ± 86	420 ± 92	0.6256
**AHI** (events/hour)	9.1 (2.9;18.8)	7.1 (2.7;18.6)	0.199	5.3 (2.4;18.8)	12.4 (2.7;20.4)	0.1451	12.9 (6;13.7)	5.2 (2.2;16.4)	0.5032
**Central Apnea Index** (n/hour)	0.1 (0;1.3)	0.1 (0;0.4)	0.3372	0.1 (0;0.7)	0.2 (0.1;1.1)	0.8408	0.1 (0;2.2)	0 (0;0.1)	0.1243
**Mixed Apnea Index** (n/hour)	0 (0;0.3)	0 (0;0.6)	0.092	0 (0;0.3)	0.1 (0;1.3)	0.2298	0 (0;0.3)	0 (0;0.2)	0.1628
**Obstructive Apnea Index** (n/hour)	1.4 (0.2;2.9)	0.8 (0.2;6.3)	0.3748	1.3 (0.1;2.9)	1 (0.3;6.3)	0.7798	1.2 (0.4;4.1)	0.3 (0;3.2)	0.3669
**Hypopnea Index** (n/hour)	3.8 (1.7;7.7)	4.8 (1.5;8.9)	0.183	3.1 (1.1;4.7)	5.9 (2.2;10.1)	0.9863	5.8 (2.9;10.5)	3.8 (0.9;6.3)	**0.0346**
**Mean SaO**_**2**_ (%)	93.2 ± 1.4	92.6 ± 2.4	0.6919	93.6 ± 1.4	92.5 ± 2.9	0.3018	92.9 ± 1.4	92.7 ± 1.7	0.8743
**Minimum SaO**_**2**_ (%)	82.1 ± 6.4	80.9 ± 9.3	0.9554	84.6 ± 4.6	80.5 ± 9.5	0.1654	80.6 ± 6.2	83.2 ± 6.2	0.1757
**Average SaO**_**2**_ **nadir** (%)	4.9 ± 1.4	5.1 ± 1.7	0.9753	4.5 ± 1.1	5.3 ± 1.8	0.2735	5.3 ± 1.7	4.8 ± 1.4	0.715
**Time at SaO**_**2**_ < **90%** (min)	8.8 (1.9;24.5)	7.8 (2.5;51.1)	0.3314	6 (0.8;13.2)	8.3 (1.8;44.4)	0.3699	12.1 (2.9;28.9)	7.1 (2.8;73.1)	0.553
**Snoring time** (%)	0.3 (0;2.6)	1.2 (0;7.9)	**0.019**	1.5 (0.1;13.8)	1.8 (0;11.6)	0.0748	0.1 (0;1.6)	0 (0;1.8)	0.1665

TIB = Time In Bed; AHI = Apnea-Hypopnea Index; SaO_2_ = Oxygen Saturation in arterial blood: Time at SaO_2_ < 90% = time spent with a SaO_2_ value < 90%.

## Discussion

In spite of the limited number of patients enrolled, some conclusions about indacaterol treatment can be drawn from our study: (a) indacaterol administration was safe in chronic HF patients treated with a β-blocker; (b) indacaterol induced a minor improvement in lung mechanics but only in bisoprolol-treated patients; (c) indacaterol had no effects on alveolar-capillary membrane diffusion regardless of β-blocker treatment; (d) indacaterol increased the ventilatory response during exercise but only in the bisoprolol group with no effects on overall exercise performance; (e) no major indacaterol-induced effects were observed on sleep quality, except for a reduction in hypopneas in the carvedilol-treated group.

COPD is a frequent comorbidity burdening HF prognosis, whose treatment is often challenging because of the possible detrimental side effects of most bronchodilator therapies^18^. A particular concern regards β_2_-stimulating agents, whose use has been frequently associated with tachycardia, hyperkinetic arrhythmias, and worsening HF in cardiac patients^3,4,19^. A major result of our study is that indacaterol, a long-acting topical selective β_2_-agonist widely used for COPD treatment^20^, proved to be safe in a population of HF patients with moderate-to-severe reduced LVEF in stable clinical conditions and on optimized pharmacological treatment. Indeed, the incidence of both serious and minor adverse events was overall trivial and similar during treatment with indacaterol and placebo. Moreover, no significant differences were observed between indacaterol and placebo in cardiac arrhythmias, blood pressure, resting HR, BNP, or MLWHFQ score. Only a slightly, albeit significantly, higher value of maximum HR during Holter EKG recording was detected with indacaterol treatment in comparison with placebo, but with similar mean and minimum HR in the 24-hour period.

In HF patients, an impairment in lung mechanics has been reported, particularly in patients with severe HF^21^. The patients we studied had moderate HF with a limited reduction in standard spirometry parameters. Nevertheless, a minor improvement was observed with indacaterol, but only in bisoprolol-treated patients. We recognize, however, that the clinical relevance of this finding is questionable, although a greater effect might be hypothesized in patients with more severe HF, who have a greater impairment of lung mechanics.

The main hypothesis of our study was that indacaterol could increase lung diffusion thanks to a more efficient removal of alveolar fluids, at least in HF patients whose alveolar β_2_-receptors are still active (i.e. not blocked by carvedilol). Indeed, the role of β_2_-receptors in modulating alveolar fluid clearance has been widely documented both in healthy subjects and in HF patients^5,7,8,22–24^. Our data do neither confirm nor deny this hypothesis. As a matter of fact, indacaterol did not change lung diffusion in comparison with placebo, either in patients treated with bisoprolol or in patients treated with carvedilol. Indeed, DL_CO_, DL_NO_, D_M_, and Vc were not significantly different in indacaterol vs. placebo, independently of the β-blocker used. This observation is actually in line with those obtained by other groups, whose results were published after the beginning of our study, on the effects of acute inhalation of β_2_-agonists. In healthy subjects, Taylor NE *et al*. did not observe any significant change in D_M_ after acute administration of albuterol^21^. In a further study on HF patients, the same group observed a reduction in lung water content after acute albuterol administration, as evaluated by computed tomography imaging combined with DL_CO_/DL_NO_-derived capillary volume, but without any difference in DL_CO_, DL_NO_, D_M_, or Vc between before and after drug administration^10^. Similarly, Di Marco *et al*. did not observe any effect of acute salmeterol administration in COPD patients on DL_CO_, even though a protective effect against lung diffusion injury after acute fluid overload challenge was detected^9^. These negative observations, however, do not necessarily imply that the stimulation of alveolar β_2_-receptors does not influence alveolar-capillary membrane diffusion conductance in HF patients. In fact, even if a mild degree of bronchodilation was observed in our population in bisoprolol-treated patients after indacaterol, there is no evidence that the drug actually reaches the alveolar compartment after topic administration. Indacaterol, as well as other β_2_-agonists, has been conceived to reach bronchioles, where it exerts its bronchodilation activity, and only marginally the alveolar tissue, where it can be absorbed in the systemic circulation and exert undesired side effects. Considering the huge area of the alveolar compartment, it is possible that the amount of drug able to stimulate the alveolar β_2_-receptors was too low to induce any detectable effect on lung diffusion. Ideally, a β_2_-agonist able to reach to a relevant extent the alveolar compartment, which to our knowledge does not exist nowadays, should be tested to further investigate the possible benefit of alveolar β_2_-receptor stimulation on lung diffusion in HF patients.

Finally, indacaterol proved to increase the ventilatory response to exercise, as demonstrated by a significantly higher VE/VCO_2_ slope^25^, in patients treated with bisoprolol but not in those treated with carvedilol. This observation was at first unexpected. Indeed, ventilation depends on VCO_2_ production, dead space/tidal volume (VD/Vt) course − which in turn depends on alveolar ventilation/perfusion matching − and arterial pCO_2_ set point, which depends on chemoreceptor sensitivity, according to the following equation: VE = k × VCO_2_/[PaCO_2_ × (1 − VD/Vt)]^26^. Peak VCO_2_ was not significantly modified by indacaterol either in bisoprolol- or in carvedilol-treated patients. VD/Vt could not be reliably calculated during exercise or at the peak of exercise in our study because of the lack of arterial samples during the tests. From a theoretical point of view, indacaterol could induce an increase in exercise VD, mostly in patients treated with a cardioselective β-blocker such as bisoprolol, assuming that bronchodilation induces a recruitment of non-perfused alveoli. However, if this were the case, an increase in maximal ventilation at the peak of exercise during indacaterol treatment, to a higher extent in the bisoprolol group, should have been observed, but it was not. Moreover, on one hand, the worsening of ventilation/perfusion mismatch during exercise is usually observed only in very severe HF patients^25^, whereas our population consisted mostly of patients with moderate left ventricular systolic dysfunction and in stable clinical conditions, and, on the other hand, the increase in FEV_1_ observed after indacaterol in the bisoprolol group (suggesting a mild degree of bronchodilation) was quantitatively negligible. An alternative, more convincing interpretation is that indacaterol increased ventilatory response to exercise by increasing chemoreceptor sensitivity to CO_2_ through a stimulation of β_2_-receptors located on chemoreceptors themselves. The observed reduction of P_ET_CO_2_ in parallel with VE/VCO_2_ increase is in line with this hypothesis. Consistently with this interpretation, this effect was only observed in bisoprolol-treated patients, and not in patients whose β_2_-receptors had been blocked by carvedilol. Indeed, chemoreceptor activity is widely modulated by hormonal and nervous stimuli, and β_2_-receptor stimulation or blockade has proved to influence chemoreceptor sensitivity, particularly in HF patients^8,27^. Indeed, there are several differences between bisoprolol and carvedilol, and carvedilol induces a more complete sympathetic block. First of all, bisoprolol is highly cardioselective, having therefore no or only trivial effects, at the usual dosages, on β_2_-receptors located in the lung, while carvedilol equally blocks β_1_ and β_2_-receptors. Moreover, carvedilol blocks both β and α-receptors, it does not upregulate cardiac β-receptors, it has central sympatholytic activity, and it is classified as a β-arrestin-biased agonist^1,28,29^. These actions could explain the different effect on chemoreceptor sensitivity, and consequently the greater ventilatory response to exercise obtained with indacaterol in the bisoprolol group. The absence of any relevant effect of indacaterol on sleep apnea, regardless of the β-blocker treatment, is not surprising, since our patients had very limited sleep abnormalities and the presence of sleep abnormalities was not among the study inclusion/exclusion criteria. It is possible, but totally unproven, that results could have been different in patients with more severe HF and sleep abnormalities.

### Study limitations

Some limitations of the study need to be acknowledged.

1. The main limitation of this study is that it was prematurely interrupted because of placebo shortage one year after the beginning of the recruitment. Consequently, the number of cases in the two β-blocker groups were different. Accordingly, more data are needed to define some of the study endpoints. However, the primary study endpoint, i.e. the effects of indacaterol on alveolar-capillary gas diffusion, is clearly negative, and it would have remained so even if all cases had been studied.
2. Carvedilol and bisoprolol groups were not created by a randomization system. This is a source of potential bias, as the clinical choice of the β-blocker is usually driven by comorbidities and in particular by the presence or absence of lung disease. However, severe lung disease was an exclusion criterion, and no significant difference was observed in patients’ clinical characteristics between carvedilol and bisoprolol groups, but for a slightly higher HR and lower LVEF in bisoprolol (see table in Supplementary Information).
3. It is totally unknown whether and how much of the inhaled drug gets to the alveoli and whether other forms of inhalation (ultrafine, different inhalator, etc.) could produce different responses.

## Conclusions

Indacaterol, a long-acting, highly β_2_-selective adrenoceptor agonist widely used for the treatment of COPD, proved to be safe in moderate-to-severe HF patients in stable clinical conditions treated with a β-blocker. An increase in ventilatory response to exercise after indacaterol was observed only in patients treated with a β_1_-selective β-blocker (bisoprolol), suggesting a role of β_2_-receptor stimulation in the regulation of the ventilatory drive. Finally, our study failed to demonstrate any effect of indacaterol treatment on lung diffusion. The inability of the indacaterol molecule to reach the alveoli and to stimulate β_2_-receptors located on the alveolar side of epithelial cells is a possible explanation.

## Supplementary information

Supplementary information.
