# Supplementary material for: Effects of β2-receptor stimulation by indacaterol in chronic heart failure treated with selective or non-selective β-blockers: a randomized trial
Source: Sci Rep. 2020 Apr 28;10:7101. doi: 10.1038/s41598-020-62644-1 (PMC7188807; doi:10.1038/s41598-020-62644-1)
Supplement: Supplementary file 1 — Supplementary information. [file 41598_2020_62644_MOESM1_ESM.pdf]

Effects of β_2-receptor stimulation by indacaterol in chronic heart failure treated with selective or non-selective β-blockers: a randomized trial.

Mauro Contini[1], MD, Emanuele Spadafora[1], PhD, Simone Barbieri[1] MSc, Paola Gugliandolo[1], CCP, Elisabetta Salvioni[1],PhD, Alessandra Magini[1], MD, Anna Apostolo[1], MD, Pietro Palermo[1], MD, Marina Alimento[1], MD, Piergiuseppe Agostoni[1,2] MD, PhD

[1] Centro Cardiologico Monzino, IRCCS, Milano, Italy

[2] Dept. Of Clinical sciences and Community health, Cardiovascular Section, University of Milano, Milano, Italy.

Brief title: Indacaterol and lung function in chronic heart failure

Corresponding Author

Piergiuseppe Agostoni, MD, PhD

Centro Cardiologico Monzino, IRCCS, Via Parea 4, 20138 Milan, Italy

Phone +39 02 58002488

Fax +39 02 58002008

E-mail: piergiuseppe.agostoni@unimi.it; piergiuseppe.agostoni@ccfm.it

Supplementary table: Comparison of patients' characteristics between bisoprolol and carvedilol groups.

	Bisoprolol	Carvedilol	p
**Number of patients**	27	17	
**Age (years)**	67±10	64±9	0,36
**Gender (Males N/%)**	18(66.7)	13(76.5)	0,73
**No smokers (N/%)**	11(40.7)	6(35.3)	0,72
**Former smokers (N/%)**	16(59.3)	11(64.7)	
**Sinus rhythm (N/%)**	22(81.5)	15(88.2)	0,68
**Atrial fibrillation (N/%)**	5(18,51)	2 (11,76)	
**Mitral regurgitation absent (N/%)**	11(42.3)	4(23.5)	0,27
**Mitral regurgitation mild (N/%)**	8(30.8)	10(58.8)	
**Mitral regurgitation moderate(N/%)**	6(23.1)	3(17.6)	
**Mitral regurgitation severe (N/%)**	1(3.8)	0(0)	
**ACE inhibitors(N/%) No**	11(40.7)	6(35.3)	0,72
**ACE inhibitors(N/%) Yes**	16(59.3)	11(64.7)	
**ATII blockers (N/%): No**	19(70.4)	13(76.5)	0,74
**ATII blockers (N/%): Yes**	8 (29,62)	4 (23,53)	
**Diuretics (N/%) No**	5(18.5)	3(17.6)	0,94
**Diuretics (N/%) Yes**	22(81.5)	14(82.4)	
**Antialdosteronics (N/%) No**	6(22.2)	4(23.5)	1
**Antialdosteronics (N/%) Yes**	21 (77,77)	13 (76,47)	
**Digitalis (N/%) No**	25(92.6)	16(94.1)	1
**Digitalis (N/%) Yes**	2 (7,40)	1 (5,88)	
**Systolic Arterial Pressure (mmHg)**	124±15	121±19	0,48
**Diastolic Arterial Pressure (mmHg)**	76±8	75±9	0,55

**Aetilogy: ischemic**	15(55.6)	8(47.1)	0,27
**Aetilogy: idiopathic**	12(44.4)	7(41.2)	
**Aetilogy: other**	0(0)	2(11.8)	
**Body mass index**	27.9±4.7	28.3±4.7	0,79
**Left Ventricular Ejection Fraction (%)**	34.6±4.6	31.2±6.8	0,05
**Rest heart rate (bpm)**	63±10	70±10	0,02
**Haemoglobin (g/dl)**	14.0±1.3	13.5±1.4	0,22
**BNP (pg/ml)**	241(140;453)	223(78;372)	0,26
**MDRD (ml/min/1.73 mq)**	72.2±19.2	71.3±24.1	0.90
**NYHA class I**	4(14.8)	3(17.6)	1
**NYHA class II**	19(70.4)	12(70.6)	
**NYHA class III**	4(14.8)	2(11.8)	
**MLWHFQ Score**	24(15;36)	35(7;50)	0,62
**FEV1 (L/min) /FEV1 (% predicted)**	2.32±0.7	2.38±0.7	0,80
**FVC (L) / FVC (% predicted)**			
**DLco (ml/min/mmHg)**	19.58±6.2	21.96±5.9	0,21
**Peak VO2 (ml/Kg/min)**	1131±369	1290±512	0,24

NYHA= NewYork Heart Association; BNP=Brain Natriuretic Peptide; MDRD= estimation of glomerular filtration rate by modification of diet in renal disease formula; MLWHF=Minnesota Living With Heart Failure; FEV1= Forced expiratory volume in the first second; FVC= forced vital capacity; DLco= lung diffusion for carbon monoxide; VO2=oxygen consumption
